# IL-12 primed CD8+ T-cells possess enhanced persistence and anti-tumor efficacy because of greater IL-7 responsiveness

**DOI:** 10.1186/2051-1426-1-S1-P17

**Published:** 2013-11-07

**Authors:** C Bryce Johnson, Bennett R May, Colleen A Cloud, Mark P Rubinstein, David J Cole

**Affiliations:** 1 Medical University of South Carolina, Charleston, SC, USA

## 

Ex vivo IL-12 priming of tumor-reactive CD8+ T-cells enhances their persistence and subsequent anti-tumor efficacy upon adoptive cell transfer (ACT) into lymphodepleted mice. Since IL-7 and IL-15 are considered critical for the persistence of adoptively transferred T-cells, we investigated the responsiveness of IL-12 primed CD8+ T-cells to these homeostatic cytokines to help elucidate the mechanisms behind their superior anti-tumor abilities. Using pmel-1 T-cell receptor transgenic mice, we found that pmel CD8+ T cells activated in the presence of IL-12 (pmelIL-12) showed much greater expansion in an irradiated (6 Gy) host compared to cells activated without IL-12 (pmelsham). This expansion was dependent on host IL-7, but not IL-15. Compared to pmelsham, pmelIL-12 demonstrated greatly enhanced in vitro IL-7 responsiveness, as measured by proliferation and phosphorylation of signaling molecules STAT5, S6 and AKT. These striking differences were not seen with IL-15. Despite not playing a major role in pmelIL-12 effector phase expansion, IL-15 was critical for maximum anti-tumor efficacy of pmelIL-12, as irradiated mice devoid of IL-15, like mice receiving IL-7 neutralizing antibody (clone M25), showed reduced survival compared to irradiated mice receiving pmelIL-12 only. This decreased tumor control likely occurred because IL-15 was needed for long-term persistence of pmelIL-12. Together, these findings suggest that IL-12 priming of CD8+ T-cells augments the efficacy of ACT protocols in part by conferring an enhanced ability to respond to IL-7, thereby enabling transferred cells to persist in the IL-7 rich post-lymphodepletion environment.

